# 1,3-Bis(2-meth­oxy­phen­yl)thio­urea

**DOI:** 10.1107/S1600536811040852

**Published:** 2011-10-08

**Authors:** Jason van Rooyen, Richard Betz, Bernardus J. A. M. van Brecht

**Affiliations:** a Nelson Mandela Metropolitan University, Summerstrand Campus, Department of Chemistry, University Way, Summerstrand, PO Box 77000, Port Elizabeth 6031, South Africa

## Abstract

In the title compound, C_15_H_16_N_2_O_2_S, the N–C(=S) bond lengths are indicative of the presence of amide-type resonance. The dihedral angles between the thio­urea unit and the attached aromatic rings are 59.80 (5) and 73.41 (4)° while the dihedral angle between the rings is 56.83 (4)°. In the crystal, inversion dimers linked by pairs of N—H⋯S hydrogen bonds occur. An N—H⋯π inter­action is observed for the second amino group. The shortest centroid–centroid distance between two aromatic systems is 4.0958 (8) Å.

## Related literature

For related structures, see: Shashidhar *et al.* (2006 ▶); Muhammed *et al.* (2007 ▶); Kuan & Tiekink (2007 ▶); Srivastava *et al.* (2010 ▶). For further synthetic details, see: Voss & Walter (1968 ▶). For graph-set analysis of hydrogen bonds, see: Etter *et al.* (1990 ▶); Bernstein *et al.* (1995 ▶). For general information about coordination chemistry, see: Gade (1998 ▶). Structures containing similar bond lengths were retrieved from the Cambridge Structural Database (Allen, 2002 ▶).
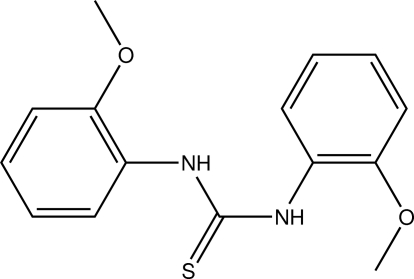


## Experimental

### 

#### Crystal data

C_15_H_16_N_2_O_2_S
*M* *_r_* = 288.36
Monoclinic, 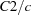

*a* = 14.3187 (8) Å
*b* = 12.8628 (7) Å
*c* = 16.1168 (10) Å
β = 103.790 (3)°
*V* = 2882.8 (3) Å^3^
*Z* = 8
Mo *K*α radiation
μ = 0.23 mm^−1^
*T* = 173 K
0.42 × 0.36 × 0.14 mm

#### Data collection

Bruker SMART CCD diffractometer
10474 measured reflections
3567 independent reflections
2765 reflections with *I* > 2σ(*I*)
*R*_int_ = 0.058

#### Refinement

*R*[*F* ^2^ > 2σ(*F* ^2^)] = 0.033
*wR*(*F* ^2^) = 0.090
*S* = 1.05
3567 reflections
191 parameters
H atoms treated by a mixture of independent and constrained refinement
Δρ_max_ = 0.25 e Å^−3^
Δρ_min_ = −0.23 e Å^−3^

### 

Data collection: *SMART* (Bruker, 1998 ▶); cell refinement: *SAINT* (Bruker, 1998 ▶); data reduction: *SAINT*; program(s) used to solve structure: *SHELXS97* (Sheldrick, 2008 ▶); program(s) used to refine structure: *SHELXL97* (Sheldrick, 2008 ▶); molecular graphics: *ORTEP-3* (Farrugia, 1997 ▶) and *Mercury* (Macrae *et al.*, 2008 ▶); software used to prepare material for publication: *SHELXL97* and *PLATON* (Spek, 2009 ▶).

## Supplementary Material

Crystal structure: contains datablock(s) I, global. DOI: 10.1107/S1600536811040852/hb6427sup1.cif

Supplementary material file. DOI: 10.1107/S1600536811040852/hb6427Isup2.cdx

Structure factors: contains datablock(s) I. DOI: 10.1107/S1600536811040852/hb6427Isup3.hkl

Supplementary material file. DOI: 10.1107/S1600536811040852/hb6427Isup4.cml

Additional supplementary materials:  crystallographic information; 3D view; checkCIF report

## Figures and Tables

**Table 1:** Hydrogen-bond geometry (Å, °) *Cg*1 is the centroid of the C11–C16 ring.

*D*—H⋯*A*	*D*—H	H⋯*A*	*D*⋯*A*	*D*—H⋯*A*
N2—H72⋯S1^i^	0.831 (16)	2.506 (17)	3.3343 (12)	174.3 (14)
N1—H71⋯*Cg*1^ii^	0.782 (16)	2.967 (18)	3.5127 (13)	129.1 (14)

Symmetry codes: (i) 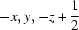; (ii) 
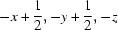
.

**Fig. 1.**
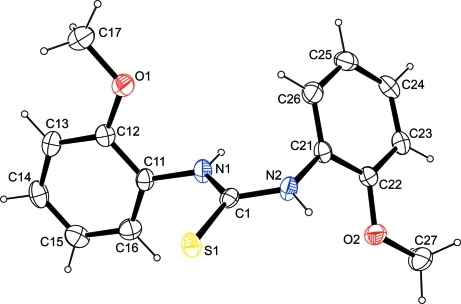
The molecular structure of the title compound, with anisotropic displacement ellipsoids (drawn at 50% probability level).

**Fig. 2.**
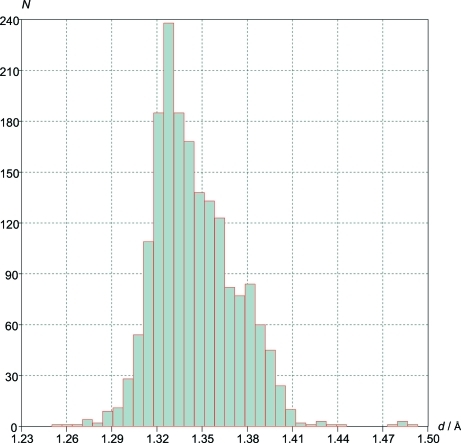
Statistical distribution of N(H)–C(=S) bond lengths in thiourea-derived amides (data based on CSD search including all deposited crystal structures up to August 2011).

**Fig. 3.**
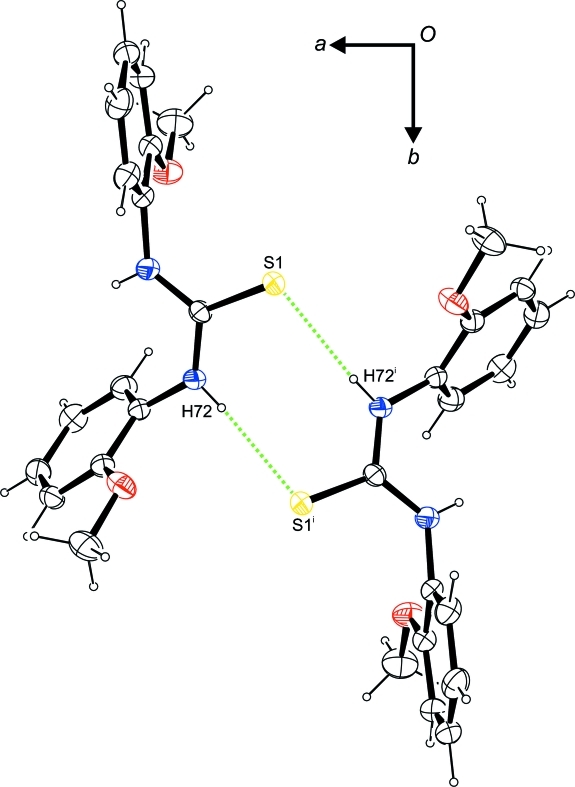
Intermolecular contacts, viewed along [0 1 0]. Symmetry operator: i -x, y, -z + 1/2.

**Fig. 4.**
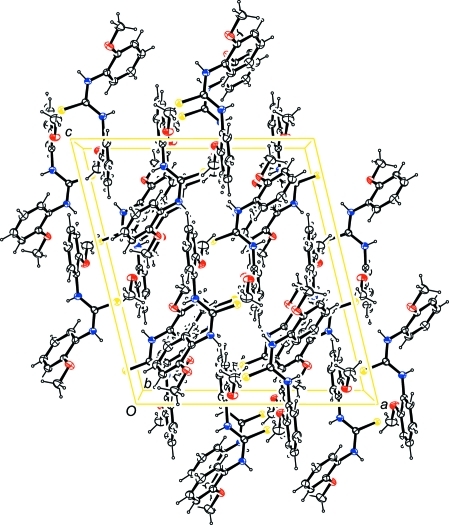
Molecular packing of the title compound, viewed along [0 1 0] (anisotropic displacement ellipsoids drawn at 50% probability level).

## supplementary crystallographic information

### Comment

Chelate ligands have found widespread use in coordination chemistry due to the enhanced thermodynamic stability of resultant coordination compounds in relation to coordination compounds exclusively applying comparable monodentate ligands (Gade, 1998). Combining different donor atoms, a molecular set-up to accomodate a large variety of metal centers of variable Lewis acidity is at hand. In this aspect, the title compound, 1,3-bis(2-methoxyphenyl)thiourea, (I), seemed of interest due to its possible use as a strictly neutral or, depending on the pH value, as an anionic or cationic ligand. In addition, due to the set-up of its functional groups, it may act as mono- or multidentate ligand offering the possibility to create chelate rings of various size. The intriguing combination of a secondary amino group, a thioketo group as well as methylether groups classifies the title compound as a highly versatile ligand. To enable comparative studies in terms of bond lengths and angles in envisioned coordination compounds, we determined the molecular and crystal structure of the title compound. Information about the crystal structure of 1,3-bis(4-methoxyphenyl)thiourea (Shashidhar *et al.*, 2006), 1,3-bis(2-methylphenyl)thiourea, (Muhammed *et al.*, 2007; Kuan & Tiekink, 2007) and 1,3-bis(phenyl)thiourea (Srivastava *et al.*, 2010) is available in the literature.

N–C=S bond lengths (*d*_N–C_: 1.3469 (15) Å and 1.3488 (16) Å, respectively) are in good agreement with values deposited for comparable compounds with the Cambridge Structural Database (Allen, 2002) and are indicative of admide-type resonance between the atoms of this entity. This finding is further corroborated by the planarity of the S=CN_2_ moiety (r.m.s. of all fitted atoms = 0.0015 Å). The aromatic substituents on the nitrogen atom adopt *syn* and *anti* conformation with respect to the sulfur atom. The least-squares planes defined by the carbon atoms of the respective phenyl rings enclose an angle of 56.83 (4) ° while the individual planes defined by the phenyl rings intersect with the least-squares plane defined by the atoms of the central S=CN_2_ moiety at angles of 59.80 (5) ° and 73.41 (4) ° (Fig. 1, Fig. 2).

In the crystal, the hydrogen atoms of the secondary amine groups participate in two different types of intermolecular interactions. While one of the protons is part of a classical hydrogen bond of the N–H···S type, the other amine group's hydrogen atom forms a contact to one of the aromatic systems. The classical hydrogen bonds connect the molecules to centrosymmetric dimers orientated approximately perpendicular to the crystallographic *b* axis. In terms of graph-set analysis (Etter *et al.*, 1990; Bernstein *et al.*, 1995), the descriptor for the classical hydrogen bonds is *R*^2^_2_(8) on the unitary level. The shortest intercentroid distance between two aromatic systems was measured at 4.0958 (8) Å (Fig. 3).

The packing of the title compound in the crystal structure is shown in Figure 4.

### Experimental

The title compound was prepared upon reacting Lawesson's reagent with the corresponding amide in analogy to a published procedure (Voss & Walter, 1968).

### Refinement

Carbon-bound H atoms were placed in calculated positions (C—H 0.95 Å) and were included in the refinement in the riding model approximation, with *U*(H) set to 1.2*U*_eq_(C). The H atoms of the methyl groups were allowed to rotate with a fixed angle around the C—C bond to best fit the experimental electron density (HFIX 137 in the *SHELX* program suite (Sheldrick, 2008), with *U*(H) set to 1.5*U*_eq_(C). Both nitrogen-bound H atoms were located on a difference Fourier map and refined freely.

### Crystal data

**Table:**

C_15_H_16_N_2_O_2_S	*Z* = 8
*M**_r_* = 288.36	*F*(000) = 1216
Monoclinic, *C*2/*c*	*D*_x_ = 1.329 Mg m^−^^3^
Hall symbol: -C 2yc	Mo *K*α radiation, λ = 0.71073 Å
*a* = 14.3187 (8) Å	µ = 0.23 mm^−^^1^
*b* = 12.8628 (7) Å	*T* = 173 K
*c* = 16.1168 (10) Å	Plate, colourless
β = 103.790 (3)°	0.42 × 0.36 × 0.14 mm
*V* = 2882.8 (3) Å^3^	

### Data collection

**Table:**

Bruker SMART CCD diffractometer	2765 reflections with *I* > 2σ(*I*)
Radiation source: fine-focus sealed tube	*R*_int_ = 0.058
graphite	θ_max_ = 28.3°, θ_min_ = 2.2°
φ and ω scans	*h* = −16→19
10474 measured reflections	*k* = −14→17
3567 independent reflections	*l* = −17→21

### Refinement

**Table:**

Refinement on *F*^2^	Primary atom site location: structure-invariant direct methods
Least-squares matrix: full	Secondary atom site location: difference Fourier map
*R*[*F*^2^ > 2σ(*F*^2^)] = 0.033	Hydrogen site location: inferred from neighbouring sites
*wR*(*F*^2^) = 0.090	H atoms treated by a mixture of independent and constrained refinement
*S* = 1.05	*w* = 1/[σ^2^(*F*_o_^2^) + (0.0437*P*)^2^ + 0.1621*P*] where *P* = (*F*_o_^2^ + 2*F*_c_^2^)/3
3567 reflections	(Δ/σ)_max_ < 0.001
191 parameters	Δρ_max_ = 0.25 e Å^−^^3^
0 restraints	Δρ_min_ = −0.23 e Å^−^^3^

### Fractional atomic coordinates and isotropic or equivalent isotropic displacement parameters (Å^2^)

**Table:**

	*x*	*y*	*z*	*U*_iso_*/*U*_eq_	
S1	−0.01726 (2)	0.20091 (3)	0.11713 (2)	0.02289 (10)	
O1	0.09218 (7)	0.42621 (7)	−0.03050 (6)	0.0323 (2)	
O2	0.24916 (6)	0.20317 (7)	0.36118 (6)	0.0291 (2)	
N1	0.14650 (8)	0.28483 (9)	0.09695 (7)	0.0251 (3)	
H71	0.1955 (12)	0.3130 (13)	0.1153 (10)	0.036 (5)*	
N2	0.12052 (7)	0.29144 (9)	0.23257 (7)	0.0232 (2)	
H72	0.0931 (11)	0.2653 (11)	0.2676 (10)	0.028 (4)*	
C1	0.08967 (8)	0.26236 (9)	0.15016 (8)	0.0197 (3)	
C11	0.12847 (8)	0.25261 (10)	0.00981 (8)	0.0223 (3)	
C12	0.10350 (9)	0.32650 (10)	−0.05553 (8)	0.0238 (3)	
C13	0.09201 (9)	0.29468 (11)	−0.14003 (9)	0.0288 (3)	
H13	0.0771	0.3444	−0.1849	0.035*	
C14	0.10227 (9)	0.19063 (12)	−0.15889 (9)	0.0307 (3)	
H14	0.0929	0.1695	−0.2168	0.037*	
C15	0.12589 (10)	0.11744 (11)	−0.09458 (9)	0.0311 (3)	
H15	0.1324	0.0463	−0.1079	0.037*	
C16	0.14008 (9)	0.14937 (11)	−0.00994 (9)	0.0281 (3)	
H16	0.1579	0.0998	0.0347	0.034*	
C17	0.06484 (12)	0.50262 (12)	−0.09648 (10)	0.0408 (4)	
H171	0.1170	0.5116	−0.1257	0.061*	
H172	0.0523	0.5689	−0.0712	0.061*	
H173	0.0066	0.4796	−0.1377	0.061*	
C21	0.20704 (8)	0.34620 (10)	0.26996 (8)	0.0215 (3)	
C22	0.27304 (8)	0.30070 (10)	0.33908 (8)	0.0218 (3)	
C23	0.35552 (9)	0.35490 (11)	0.37961 (8)	0.0257 (3)	
H23	0.3997	0.3251	0.4272	0.031*	
C24	0.37282 (9)	0.45274 (11)	0.35004 (9)	0.0287 (3)	
H24	0.4298	0.4889	0.3770	0.034*	
C25	0.30840 (10)	0.49852 (10)	0.28199 (9)	0.0299 (3)	
H25	0.3210	0.5655	0.2623	0.036*	
C26	0.22491 (9)	0.44499 (11)	0.24273 (9)	0.0267 (3)	
H26	0.1797	0.4765	0.1968	0.032*	
C27	0.31264 (10)	0.15497 (12)	0.43256 (10)	0.0358 (3)	
H271	0.3761	0.1461	0.4205	0.054*	
H272	0.2869	0.0869	0.4430	0.054*	
H273	0.3185	0.1989	0.4832	0.054*	

### Atomic displacement parameters (Å^2^)

**Table:**

	*U*^11^	*U*^22^	*U*^33^	*U*^12^	*U*^13^	*U*^23^
S1	0.01794 (15)	0.03006 (19)	0.02002 (17)	−0.00105 (12)	0.00324 (11)	−0.00245 (13)
O1	0.0424 (6)	0.0268 (5)	0.0263 (5)	0.0036 (4)	0.0056 (4)	0.0003 (4)
O2	0.0276 (5)	0.0279 (5)	0.0269 (5)	−0.0045 (4)	−0.0032 (4)	0.0055 (4)
N1	0.0220 (5)	0.0345 (7)	0.0186 (6)	−0.0060 (5)	0.0046 (4)	−0.0010 (5)
N2	0.0198 (5)	0.0333 (6)	0.0162 (5)	−0.0042 (4)	0.0035 (4)	0.0021 (5)
C1	0.0190 (5)	0.0199 (6)	0.0193 (6)	0.0044 (4)	0.0026 (5)	0.0035 (5)
C11	0.0195 (5)	0.0302 (7)	0.0183 (6)	−0.0015 (5)	0.0065 (5)	−0.0004 (6)
C12	0.0218 (6)	0.0275 (7)	0.0225 (7)	0.0000 (5)	0.0059 (5)	−0.0017 (5)
C13	0.0274 (6)	0.0381 (8)	0.0206 (7)	0.0024 (6)	0.0052 (5)	0.0017 (6)
C14	0.0256 (6)	0.0439 (9)	0.0233 (7)	−0.0013 (6)	0.0072 (5)	−0.0096 (6)
C15	0.0312 (7)	0.0299 (8)	0.0354 (8)	−0.0016 (5)	0.0144 (6)	−0.0063 (6)
C16	0.0277 (6)	0.0292 (7)	0.0296 (8)	−0.0007 (5)	0.0112 (5)	0.0018 (6)
C17	0.0526 (9)	0.0313 (8)	0.0366 (9)	0.0041 (7)	0.0067 (7)	0.0071 (7)
C21	0.0201 (5)	0.0267 (7)	0.0181 (6)	−0.0015 (5)	0.0056 (5)	−0.0035 (5)
C22	0.0225 (6)	0.0226 (6)	0.0205 (6)	−0.0003 (5)	0.0056 (5)	−0.0025 (5)
C23	0.0230 (6)	0.0308 (8)	0.0214 (7)	0.0010 (5)	0.0016 (5)	−0.0049 (6)
C24	0.0267 (6)	0.0289 (8)	0.0306 (8)	−0.0052 (5)	0.0074 (6)	−0.0120 (6)
C25	0.0367 (7)	0.0210 (7)	0.0342 (8)	−0.0026 (5)	0.0129 (6)	−0.0032 (6)
C26	0.0293 (6)	0.0263 (7)	0.0246 (7)	0.0035 (5)	0.0067 (5)	−0.0002 (6)
C27	0.0371 (7)	0.0326 (8)	0.0321 (8)	0.0016 (6)	−0.0027 (6)	0.0081 (7)

### Geometric parameters (Å, °)

**Table:**

S1—C1	1.6923 (12)	C15—H15	0.9500
O1—C12	1.3658 (16)	C16—H16	0.9500
O1—C17	1.4324 (17)	C17—H171	0.9800
O2—C22	1.3695 (15)	C17—H172	0.9800
O2—C27	1.4266 (16)	C17—H173	0.9800
N1—C1	1.3469 (15)	C21—C26	1.3879 (19)
N1—C11	1.4275 (16)	C21—C22	1.4053 (17)
N1—H71	0.782 (16)	C22—C23	1.3928 (17)
N2—C1	1.3488 (16)	C23—C24	1.3887 (19)
N2—C21	1.4277 (15)	C23—H23	0.9500
N2—H72	0.831 (16)	C24—C25	1.385 (2)
C11—C16	1.3848 (18)	C24—H24	0.9500
C11—C12	1.4003 (18)	C25—C26	1.3939 (19)
C12—C13	1.3937 (19)	C25—H25	0.9500
C13—C14	1.388 (2)	C26—H26	0.9500
C13—H13	0.9500	C27—H271	0.9800
C14—C15	1.381 (2)	C27—H272	0.9800
C14—H14	0.9500	C27—H273	0.9800
C15—C16	1.392 (2)		
			
C12—O1—C17	117.11 (11)	O1—C17—H172	109.5
C22—O2—C27	117.21 (10)	H171—C17—H172	109.5
C1—N1—C11	124.69 (11)	O1—C17—H173	109.5
C1—N1—H71	118.9 (12)	H171—C17—H173	109.5
C11—N1—H71	116.2 (12)	H172—C17—H173	109.5
C1—N2—C21	126.87 (11)	C26—C21—C22	119.49 (11)
C1—N2—H72	117.3 (10)	C26—C21—N2	121.67 (11)
C21—N2—H72	114.6 (10)	C22—C21—N2	118.75 (11)
N1—C1—N2	117.50 (11)	O2—C22—C23	124.96 (12)
N1—C1—S1	122.59 (10)	O2—C22—C21	115.17 (11)
N2—C1—S1	119.91 (9)	C23—C22—C21	119.87 (12)
C16—C11—C12	120.07 (12)	C24—C23—C22	119.56 (12)
C16—C11—N1	120.07 (12)	C24—C23—H23	120.2
C12—C11—N1	119.79 (12)	C22—C23—H23	120.2
O1—C12—C13	124.73 (12)	C25—C24—C23	121.17 (12)
O1—C12—C11	116.24 (11)	C25—C24—H24	119.4
C13—C12—C11	119.02 (13)	C23—C24—H24	119.4
C14—C13—C12	120.20 (13)	C24—C25—C26	119.12 (13)
C14—C13—H13	119.9	C24—C25—H25	120.4
C12—C13—H13	119.9	C26—C25—H25	120.4
C15—C14—C13	120.84 (13)	C21—C26—C25	120.76 (13)
C15—C14—H14	119.6	C21—C26—H26	119.6
C13—C14—H14	119.6	C25—C26—H26	119.6
C14—C15—C16	119.12 (13)	O2—C27—H271	109.5
C14—C15—H15	120.4	O2—C27—H272	109.5
C16—C15—H15	120.4	H271—C27—H272	109.5
C11—C16—C15	120.70 (13)	O2—C27—H273	109.5
C11—C16—H16	119.6	H271—C27—H273	109.5
C15—C16—H16	119.6	H272—C27—H273	109.5
O1—C17—H171	109.5		
			
C11—N1—C1—N2	174.85 (11)	N1—C11—C16—C15	−177.83 (11)
C11—N1—C1—S1	−5.62 (18)	C14—C15—C16—C11	1.57 (19)
C21—N2—C1—N1	1.47 (19)	C1—N2—C21—C26	60.94 (18)
C21—N2—C1—S1	−178.08 (10)	C1—N2—C21—C22	−122.51 (14)
C1—N1—C11—C16	−71.93 (16)	C27—O2—C22—C23	1.70 (18)
C1—N1—C11—C12	111.13 (14)	C27—O2—C22—C21	−178.28 (11)
C17—O1—C12—C13	1.17 (18)	C26—C21—C22—O2	−179.98 (11)
C17—O1—C12—C11	−178.84 (12)	N2—C21—C22—O2	3.39 (16)
C16—C11—C12—O1	179.11 (11)	C26—C21—C22—C23	0.04 (18)
N1—C11—C12—O1	−3.95 (16)	N2—C21—C22—C23	−176.59 (11)
C16—C11—C12—C13	−0.90 (17)	O2—C22—C23—C24	178.63 (11)
N1—C11—C12—C13	176.04 (11)	C21—C22—C23—C24	−1.39 (19)
O1—C12—C13—C14	−177.99 (12)	C22—C23—C24—C25	1.41 (19)
C11—C12—C13—C14	2.02 (18)	C23—C24—C25—C26	−0.1 (2)
C12—C13—C14—C15	−1.4 (2)	C22—C21—C26—C25	1.34 (19)
C13—C14—C15—C16	−0.4 (2)	N2—C21—C26—C25	177.86 (12)
C12—C11—C16—C15	−0.89 (18)	C24—C25—C26—C21	−1.3 (2)

### Hydrogen-bond geometry (Å, °)

**Table:**

Cg1 is the centroid of the C11–C16 ring.

**Table:**

*D*—H···*A*	*D*—H	H···*A*	*D*···*A*	*D*—H···*A*
N2—H72···S1^i^	0.831 (16)	2.506 (17)	3.3343 (12)	174.3 (14)
N1—H71···Cg1^ii^	0.782 (16)	2.967 (18)	3.5127 (13)	129.1 (14)

Symmetry codes: (i) −*x*, *y*, −*z*+1/2; (ii) −*x*+1/2, −*y*+1/2, −*z*.
